# HPV infection among rural American Indian women and urban white women in South Dakota: an HPV prevalence study

**DOI:** 10.1186/1471-2334-11-252

**Published:** 2011-09-24

**Authors:** Delf C Schmidt-Grimminger, Maria C Bell, Clemma J Muller, Diane M Maher, Subhash C Chauhan, Dedra S Buchwald

**Affiliations:** 1 Avera Cancer Institute, Sioux Falls, SD, USA; 2 Department of Obstetrics and Gynecology, Sanford School of Medicine of the University of South Dakota, Sioux Falls, SD, USA; 3 Sanford Research, Sioux Falls, SD, USA; 4 Partnerships for Native Health, Department of Medicine, University of Washington, Seattle, WA, USA

**Keywords:** cervical cancer, Pap screening, HPV genotypes, American Indians, health disparities, human papillomavirus, types

## Abstract

**Background:**

High-risk strains of human papillomavirus (HPV) cause cervical cancer. American Indian (AI) women in the Northern Plains of the U.S. have significantly higher incidence and mortality rates for cervical cancer than White women in the same geographical area. We compared HPV prevalence, patterns of HPV types, and infection with multiple HPV types in AI and White women living in South Dakota, U.S.

**Methods:**

We analyzed the HPV status of cervical samples collected in 2006-2008 from women aged 18-65 years who attended two rural AI reservation clinics (*n *= 235) or an urban clinic in the same area serving mostly White women (*n *= 246). Data collection occurred before HPV vaccination was available to study participants. HPV DNA was amplified by using the L1 consensus primer system and an HPV Linear Array detection assay to identify HPV types. We used chi-square tests to compare HPV variables, with percentages standardized by age and lifetime number of sexual partners.

**Results:**

Compared to White women, AI women were younger (*p *= 0.01) and reported more sexual partners (*p *< 0.001). A lower percentage of AI women tested negative for HPV infection compared to Whites (58% [95% CI = 51-65] vs. 77% [95% CI = 71-82]; *p *< 0.001), and a higher percentage of AI women were infected by oncogenic types (30% [95% CI = 25-36] vs. 16% [95% CI = 11-21]; *p *= 0.001). Infections among AI women showed a wider variety and very different pattern of HPV types, including a higher prevalence of mixed HPV infections (19% [95% CI = 26-38] vs. 7% [95% CI = 4-11]; *p *= 0.001). AI women had a higher percentage of HPV infections that were not preventable by HPV vaccination (32% [95% CI = 26-38] vs. 15% [95% CI = 11-21]; *p *< 0.001).

**Conclusions:**

A higher HPV burden and a different HPV genotyping profile may contribute to the high rate of cervical cancer among AI women.

## Background

Cervical cancer is the second most common cause of cancer-related deaths in women worldwide [1] and a leading cause of cancer mortality among American Indian (AI) women [2,3]. Compared to other U.S. racial and ethnic groups, AI women experience striking health disparities in relation to cervical cancer, including a higher prevalence [4-9], a more rapidly increasing incidence [10,11], and poorer survival [12,13]. The Aberdeen Area Indian Health Service, which encompasses 16 different tribes located in South Dakota, North Dakota, Nebraska, and Iowa, reported an age-adjusted cervical cancer mortality rate of 4.3/100,000 from 1996 to 1998 [14], almost twice the rate reported in the general U.S. population [15]. Our previous work found a high prevalence of human papillomavirus (HPV) infection among AI women residing on reservations in the same geographic region, and suggested that types other than HPV-16 and HPV-18 comprised a substantial proportion of oncogenic HPV infections [16]. In a recent study of more than 9,600 U.S. women, the overall prevalence of high-risk HPV types among AI and Alaska Native women (representing 2% of the total sample) was 25% [17], which is substantially higher than the general U.S. population prevalence of 15% [18].

Infection with HPV, especially HPV-16 and HPV-18, is the most common cause of cervical cancer [19]. Preventing HPV infection is critical to public health efforts to reduce precancerous lesions and cervical cancer incidence and mortality. In 2007, the U.S. Food and Drug Administration approved the first vaccine to prevent infection by the most common oncogenic HPV types present in the general U.S. population; a second HPV vaccine was approved in 2009. Despite striking cervical cancer disparities, little is known about patterns of HPV infection in AI communities that can inform the adequacy of prevention provided for AI women by existing HPV vaccines. We therefore examined women seen in two rural AI reservation clinics and one urban clinic serving primarily White women in the Northern Plains. Our aims were to 1) compare HPV prevalence and patterns between the AI and White clinic samples, and 2) compare proportions of women in each group who were infected by HPV types that can be prevented by existing vaccines.

## Methods

### Setting

This study was conducted in two AI reservation clinics in South Dakota operated by the Indian Health Service, and in one urban clinic serving primarily White women located in Sioux Falls, South Dakota. The Indian Health Service is an agency of the U.S. Department of Health and Human Services that provides healthcare to AIs and Alaska Natives. Both reservation sites are large, rural, and extremely economically disadvantaged. According to 2004 data, 38,000 tribal members were living within reservation boundaries at Site 1, and in fiscal year 2002, 4,406 women between the ages of 15 and 64 received services at the Indian Health Service unit at this site [20]. Site 2 had 21,245 tribal members living within reservation boundaries in 2004 [21], and in fiscal year 2002, 3,100 women between the ages of 15 and 64 received services at the Indian Health Service unit at this site. Of note, the high school dropout rate in Site 1 is over 70%, and the teacher turnover rate is 800% that of the U.S. national average [22]. In contrast, in Site 2, the tribe implemented a program that increased graduation rates from 48% to 72% at the main public high school [23].

The urban site in Sioux Falls is a multi-specialty obstetrics and gynecology clinic serving South Dakota and parts of Minnesota, Iowa, and Nebraska, with approximately 30,000 outpatient visits annually. In 2003, the population of Sioux Falls was 133,834, and only 6% of residents lived below the poverty line [24]. The urban population is predominantly White (92%), with small numbers of Latino (2%), African American (2%), Asian (1%), and AI (2%) residents [25]. Only 5% of adults lack a high school education or equivalent [26].

Of note, 99% of women attending the two reservation clinics self-identified as AI and 96% of women from the urban clinic self-identified as White. Hereafter, we refer to the rural sample (Site 1 and Site 2) as AI and the urban sample as White.

Before enrollment, all women signed an informed consent form to participate in this study. Both this project and the associated consent forms were approved by the institutional review boards of the University of South Dakota and the University of Washington, as well as by the Aberdeen Area Tribal Review Board and the individual participating tribes. Tribal approval included review by the tribal Health and Human Service Committees and formal resolutions from the tribal councils.

### Participants

Potential candidates for this study included all sexually active women with an intact uterus, aged 18-65 years, who presented for annual gynecologic examinations or other non-malignancy related reasons. Data collection for all White women took place before the Gardasil vaccine was approved in 2007, so that no White participants had prior HPV vaccination. Data collection for AI women took place over a longer time frame because of recruitment issues, so that some AI participants were sampled after vaccine approval. However, the Indian Health Service did not pay for HPV vaccination during this time period, and the women recruited for our study were older than the age range initially targeted for the vaccine (11-12 years) [27], so we are confident that no AI participants had prior HPV vaccination.

Two of the authors trained all clinic staff and providers. Training consisted of a formal explanation of the project and the procedures for collecting and handling study samples, followed by direct observation of interactions with patients. Given budget constraints, clinical commitments, and other assignments, study staff were not always available at clinic sites to enroll patients. When present in the clinics, study staff reviewed patients' charts to assess eligibility, and eligible women were then invited by participating physicians to enroll in the study. Pregnant women were included if their enrolling physicians noted no health risk or contraindication, such as preterm labor or vaginal bleeding. In most cases, a Pap test was obtained first, followed by cervical sampling and completion of surveys. Surveys were administered by a trained staff member in a private setting.

### Oncogenic HPV types

All HPV genotypes that cause cervical cancer belong to the Alpha genus. These include HPV-51 (Alpha 5); HPV-56 and HPV-66 (Alpha 6); HPV-18, HPV-39, HPV-45, and HPV-59 (Alpha 7); and HPV-16, HPV-31, HPV-33, HPV-35, HPV-52, and HPV-58 (Alpha 9) [28,29]. Of note, the Alpha 7 and Alpha 9 types are most frequently implicated in cervical cancer cases worldwide, although all types in the Alpha genus have been implicated.

### HPV Vaccines

Two HPV vaccines have been approved in the U.S. The first, Gardasil, was approved in 2007 and prevents infection by HPV types 6, 11, 16, and 18. The second, Cervarix, was approved in 2009 and prevents infection by HPV types 16 and 18, with prescription information citing a *post hoc *analysis that showed efficacy against HPV-31 related CIN 2+ lesions. Our analysis focused only on the four HPV types prevented by Gardasil. Because of scientific uncertainty, we did not consider any potential HPV protection through the reported "cross protection" phenomenon [30].

### Sample Collection and Assay Methods

The study was conducted between December 2006 and November 2008. At each site, four to seven providers collected study samples. Cervical swabs were collected from the cervix under visualization. Each cervical sample was immediately chilled on ice and later cataloged and frozen at -80° C. Samples from the urban clinic were collected daily and taken to the study laboratory. Samples from the two Indian Health Service units were batch-shipped on ice overnight to the same laboratory. After all samples were collected, they were processed by the same technician, and HPV analysis was conducted under the same laboratory conditions with HPV test kits bearing the same lot number.

After the DNA samples were defrosted, a digestion solution was added to 1.0 ml of sample to achieve a concentration of 200 micrograms of proteinase K and 0.1% laureth-12, then incubated for 1 hour at 56° C. DNA was precipitated from a 150 microliters aliquot of digested material overnight at -20° C in 1.0 ml of absolute ethanol containing 75 microliters of 5.0 M ammonia acetate. After the precipitated DNA was centrifuged at 13,000 g for 30 minutes, supernatant was aspirated and the remaining DNA was dried overnight at room temperature. The pellet was resuspended in 20 microliters of 10 mM Tris and 1 mM EDTA, then incubated for 15 minutes at 95° C. The integrity of the extracted DNA was confirmed by a standard 1% agarose gel electrophoresis followed by staining with ethidium bromide.

The DNA extracts were stored at -80° C until amplification by PCR using the L1 consensus primer system and processed for HPV genotyping as described in a previous study [16]. Briefly, 20 microliters of PCR product were separated on a 2% agarose gel to confirm amplification of internal controls (Β-globin) and the HPV L1 gene. HPV genotyping strips (Roche Diagnostics, Indianapolis, IN) were used to detect 37 high- and low-risk HPV genotypes, including high-risk genotypes that could progress to cervical cancer. Non-oncogenic HPV genotypes included 6, 11, 26, 40, 42, 53, 54, 55, 61, 62, 64, 69, 71, 72, 81, 82, 83, 84, IS39, and CP6108. Oncogenic or probably oncogenic HPV genotypes included 16, 18, 31, 33, 35, 39, 45, 51, 52, 56, 58, 59, 66, 67, 68, 70, and 73. Reactions were amplified with a Biorad system according to published instructions. Seventy-five microliters of denatured, biotinylated amplified product were placed with genotyping strips into individual wells of the typing trays and covered with hybridization buffer. After HPV DNA detection, individual strips were rinsed in deionized water and stored in citrate buffer. Interpretation was based on a labeled acetate overlay with lines indicating the position of each probe relative to a reference mark on the strip denoting high- and low-risk types. Two independent investigators interpreted the data, with discrepancies resolved by a third investigator.

### Measures

We categorized age in years (18-24, 25-34, 35-44, 45-54, 55-65) and lifetime number of sexual partners (1, 2-3, 4-5, 6-9, ≥10), after checking to ensure that the categorized variables performed as well as the continuous variables in the statistical analysis. We chose this approach to simplify presentation of standardized descriptive statistics, and to allow for easier modeling of potential non-linear associations between age and HPV infection. We defined infection status as not infected, infected by a single HPV type, or infected by multiple HPV types. We measured oncogenic infection with three separate and independent variables indicating Alpha 7 infection (oncogenic HPV types 16, 31, 33, 35, 52, 58, or 67), Alpha 9 infection (oncogenic HPV types 18, 39, 45, 59, 68, or 70), and infection by oncogenic HPV types not classified as Alpha 7 or Alpha 9 (HPV types 51, 56, 66, 73, or 82). Based on this information we created a global variable indicating infection by at least one oncogenic HPV type.

We used two sets of variables to compare each woman's HPV infection profile to the four HPV types prevented by the Gardasil vaccine (types 6, 11, 16, and 18). First, we created four binary indicators of infection by each of the four types. Next, we created a single composite variable to represent hypothetical protection by Gardasil, assuming a counterfactual scenario in which each woman received vaccination before HPV exposure. This variable comprised four mutually exclusive categories, defined as 1) not infected by any HPV type, 2) no prevention (infected *only *by HPV types not prevented by the vaccine), 3) partial prevention (infected by at least one of HPV types 6, 11, 16, or 18 *and *at least one other HPV type not prevented by the vaccine), or 4) full prevention (infected *only *by HPV types 6, 11, 16, or 18).

### Statistical Analysis

Preliminary data analyses indicated that the two AI groups were sufficiently similar in relation to all study variables to combine them into a single sample for this analysis. To examine our first study aim, we calculated percentages to describe the distribution of age, lifetime number of sexual partners, HPV infection status, and oncogenic infection measures separately by clinic population (reservation vs. urban), with the chi-square test to compare the distributions. We anticipated that age and number of sexual partners, which are known risk factors for HPV infection, might be differently distributed between AI and White participants. Therefore, we adjusted HPV infection status and oncogenic infection percentages for these variables by using direct standardization, and we present them with 95% confidence intervals (CI). We also calculated the prevalence of each HPV infection status category by age group, using a non-parametric test for trend to evaluate the correlation of age and any HPV infection separately for each clinic population. To visually represent HPV prevalence patterns, we created a bar chart showing the percentage of patients from each clinic population who tested positive for each of 36 different HPV types.

For our second study aim, we calculated the percentage of women who were infected by each of the four HPV types (6, 11, 16, and 18) that are prevented by Gardasil. We calculated percentages to compare the distribution of hypothetical vaccine prevention (not infected, infection not prevented by the vaccine, infection partially prevented by the vaccine, infection completely prevented by the vaccine) by clinic population. All percentage values were adjusted for age and number of sexual partners, and are presented with 95% CI, with the chi-square test to compare distributions between the two populations.

All analyses were performed by using Stata version 10 [31]. For chi-square tests, we considered an alpha of 0.05 as the threshold for statistical significance.

## Results

We collected cervical samples from 258 AI women (104 and 154 women at Sites 1 and 2, respectively) and from 252 White women at the urban clinic. Participation rates exceeded 90%, with the total number of refusals ranging from four to seven among the three clinic sites. Some faint positive HPV results required adjudication by a third reader, but all positive results were typable. After excluding three women with missing data for HPV status and 26 women with missing data for age or number of sexual partners, data on 481 women (AI *n *= 235, White *n *= 246) were available for analysis. The vast majority of study participants were recruited during annual gynecological examinations. All participants had health insurance of some kind. For AI women, healthcare was provided and paid for by the Indian Health Service; the White women were covered by private or government-sponsored insurance (e.g., Medicaid).

As shown in Table 1, AI women were typically younger (*p *= 0.01) and reported a higher lifetime number of sexual partners (*p *< 0.001) than their White counterparts.

**Table 1:** Distribution of risk factors and HPV prevalence by clinic site

	American Indian	White	
	(*n *= 235)	(*n *= 246)	
Risk factors	%	%	***Χ***^***2 ***^***p-value***
Age, *years*			
18 - 24	26	13	0.01
25 - 34	31	33	
35 - 44	25	29	
45 - 54	12	18	
55 - 65	6	8	
Sexual partners			
1	7	23	< 0.001
2 - 3	24	29	
4 - 5	12	12	
6 - 9	33	24	
≥ 10	24	12	
**HPV prevalence**^a^	**% (95% CI)**	**% (95% CI)**	***Χ***^***2***^***p-value***
Infection status			
Not infected	58 (51-65)	77 (71-82)	< 0.001
Infected by single HPV type	23 (18-30)	16 (12-21)	
Infected by multiple HPV types	19 (15-24)	7 (4-11)	
Alpha 7 oncogenic infection^b^	12 (9-16)	8 (5-13)	0.03
Alpha 9 oncogenic infection^c^	18 (13-24)	9 (6-14)	< 0.001
Other oncogenic infection^d^	8 (5-11)	2 (1-4)	0.001
Any oncogenic infection	30 (25-36)	16 (11-21)	< 0.001

^a ^Prevalence estimates are standardized by age and number of sexual partners; ^b ^HPV types 16, 31, 33, 35, 52, 58, and 67; ^c ^HPV types 18, 39, 45, 59, 68, and 70; ^d ^HPV types 51, 56, 66, 73, 82.

**Figure 1:**
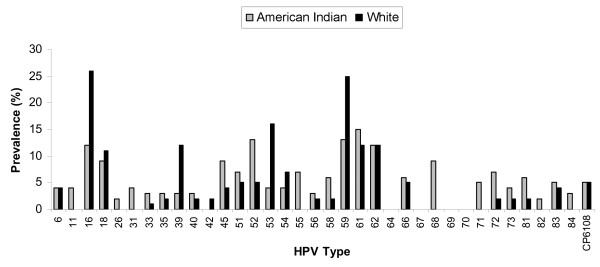
**Unstandardized prevalence of each HPV type among American Indian and White women in South Dakota**.

One hundred thirteen AI women and 57 White women tested positive for HPV. After standardization for age and number of sexual partners, prevalent HPV infection status differed between the two groups (*p *< 0.001). Overall HPV infection estimates were 42% for AI women and 23% for White women, with prevalence of multiple HPV infection nearly three times higher in AI women than in White women (19% vs. 7%). AI women also had higher standardized prevalence estimates for infection by Alpha 7, Alpha 9, and other oncogenic types, as well as nearly double the prevalence of infection by any oncogenic HPV type, compared to White women (30% vs. 16%, *p *< 0.001). In addition, AI and White women exhibited different distributions of HPV infection across age categories (Table 2). Among AI women, prevalent infection decreased from the youngest to the oldest age groups (*p*_*trend *_< 0.001), whereas no age trend was evident among White women (*p*_*trend *_= 0.23). Figure 1 displays the prevalence for each of 36 HPV types. Overall, AI women exhibited a wider variety of HPV infections, with seven HPV types detected only in AI women. In contrast, White women showed less variation in prevalent infections, with just one HPV type detected only in White women.

**Table 2:** Prevalence of single and multiple HPV infection by age category for American Indian and White women

	Infection Status					
	
	None		Single HPV Type		Multiple HPV Types	
**American Indian**^**a**^	%	(95% CI)	%	(95% CI)	%	(95% CI)
Age, *years*						
18 - 24	28	(18-40)	31	(21-44)	41	(29-54)
25 - 34	48	(37-59)	33	(23-45)	19	(12-30)
35 - 44	69	(56-80)	19	(11-31)	12	(6-23)
45 - 54	69	(50-83)	14	(5-32)	17	(7-36)
55 - 65	71	(44-89)	14	(4-43)	14	(4-43)
						
**White**^**b**^	**%**	**(95% CI)**	**%**	**(95% CI)**	**%**	**(95% CI)**
Age, *years*						
18 - 24	75	(57-87)	9	(3-25)	16	(7-33)
25 - 34	73	(63-82)	16	(9-26)	11	(6-20)
35 - 44	79	(67-87)	16	(9-26)	6	(2-14)
45 - 54	77	(62-87)	21	(11-34)	2	(0-15)
55 - 65	90	(66-97)	11	(3-34)	0	--

Results are unstandardized and presented as prevalence estimates with 95% CIs.

^a ^Any HPV infection non-parametric test for trend *p *< 0.001

^b ^Any HPV infection non-parametric test for trend *p *= 0.29

Table 3 shows the percentages of women infected with each of the HPV types prevented by the Gardasil vaccine, standardized for age and number of sexual partners. There was no statistically significant difference in the percentages of women infected by HPV types 6 or 16, but significantly more AI women were infected by HPV-11 (*p *= 0.03) and HPV-18 (*p *= 0.02). The two clinic populations differed significantly with regard to the prevalence of infections by HPV types that are not prevented by Gardasil (*p *< 0.001). The AI group had a strikingly higher percentage of women with HPV infections that would not have been prevented at all by the vaccine (32% vs. 15%), and a higher percentage of women with infections that would have been only partially prevented by the vaccine (5% vs. 2%).

**Table 3:** Prevalence of HPV types prevented by the Gardasil HPV vaccine and hypothetical vaccine protection against prevalent infections^a^

	American Indian		White	
	(*n *= 235)		(*n *= 246)	
	
	%	(95% CI)	%	(95% CI)
**Infected by HPV types prevented by Gardasil vaccine**				
Type 6	1	(0-3)	1	(0-2)
Type 11	1	(1-3)	0	--
Type 16	5	(3-8)	6	(3-10)
Type 18	5	(3-10)	1	(1-3)
**Hypothetical protection**^**a **^**by Gardasil against prevalent HPV infection**				
Not applicable (no infection)	58	(51-65)	77	(71-82)
No protection	32	(26-38)	15	(11-21)
Partial protection	5	(3-8)	2	(1-4)
Full protection	5	(3-10)	6	(3-10)

Prevalence estimates are standardized by age and lifetime number of sexual partners.

^a ^Presumed protection against infection, assuming a counterfactual scenario in which each woman had been vaccinated before HPV exposure.

## Discussion

The primary aims of this study were to examine the prevalence of HPV infection and the distribution of HPV genotypes, as well as the potential benefit of HPV vaccination, in two distinct South Dakota populations. We found that the prevalence of HPV infection among AI women was more than twice that of White women in our samples. Because we are extremely confident that none of the study participants had prior Gardasil vaccination, these differences cannot be attributed, even in part, to discrepancies in vaccine administration or uptake. Increasing age was negatively correlated with HPV infection among AI women, whereas no such trend was detected among the White sample. These findings suggest that efforts to prevent HPV infection must be tailored to the disease burden, the specific HPV prevalence patterns, and the educational and social background of the target community.

We previously reported that 22% of AI women in a Northern Plains service unit had HPV infections, a prevalence considerably higher than the 7% observed among AI women in New Mexico [4] and comparable to the 21% documented for Alaska Native women [5]. In a recent study of a large and diverse population of U.S. women, Datta and colleagues reported the prevalence of high-risk HPV infection among AI and Alaska Native women as 25% [17]. High-risk types were determined by the Hybrid Capture 2 assay (Digene, Gaithersburg, MD), which returns a positive result in the presence of any of 13 high-risk HPV types (16, 18, 31, 33, 35, 39, 45, 51, 52, 56, 58, 59, or 68). Unlike the present study, however, Datta and colleagues did not identify individual HPV types, and their sample included fewer than 180 AI women recruited nationwide.

The disparity in HPV prevalence between AI and White women residing in the Northern Plains may contribute to the disproportionately high rate of cervical cancer in the AI population. The Northern Plains incidence rate for cervical cancer in AI women is 11.3 per 100,000, which is 1.5 times higher than in non-Hispanic White women (7.5 per 100,000). Recent publications have documented an incidence of cervical cancer among AI women in South Dakota as high as 16.2/100,000, compared to 6.1/100,000 among non-Hispanic White women [9], and an age-adjusted cervical cancer mortality in the Dakotas nearly twice the national average (4.5 per 100,000 vs. 2.7 per 100,000) [8].

We also observed different patterns of HPV infection in our two study populations. Overall, AI women showed more variation in prevalent HPV types, and significantly more AI women were infected by HPV types that would not have been prevented by current HPV vaccines. Previous genotyping studies conducted in predominantly White populations have documented that HPV-16 and HPV-18 are implicated in up to 70% of cervical cancer cases in the U.S and worldwide [32,33], but virtually no AI women participated in these studies. Although vaccination is still in order, our results suggest that the protective benefit conferred by current HPV vaccines might be less for some AI women than for their White counterparts. Of note, recent evidence suggests that despite receiving HPV vaccination, some women still develop cervical cancer, likely unrelated to HPV-16 or HPV-18 [34]. Among AI women in the present study, the diversity of oncogenic types that are not preventable by existing vaccines might further contribute to the high rates of cervical cancer previously observed among AI women in the Northern Plains.

Our findings have substantial relevance to public health efforts aimed at improving cervical cancer screening among AI women. The "All Women Count" cancer screening program funded by the State of South Dakota covers Pap testing for eligible women under the age of 30, but likely misses many women with HPV and cervical dysplasia [35]. Notably, the National Breast and Cervical Cancer Early Detection Program found that AI women more often reported never having a Pap test than their non-AI counterparts, and also had the highest proportion (4.4%) of abnormal Pap tests [36]. Although our findings need to be replicated in other AI communities, they raise the question of whether the Indian Health Service might consider universal HPV screening for selected service units.

This study is limited in several ways. First, because the study population was a convenience sample drawn from the local service units, our findings may not be pertinent to all AI women living in the Northern Plains of the U.S., and cannot be generalized to other rural or urban populations. Nevertheless, we had an exceptionally high participation rate, and we believe that our samples are an accurate representation of the patient populations, both AI and White, at all clinic sites. Of note, we have additional, unpublished results from 212 AI women living in Montana (ages 16-65), suggesting an HPV prevalence of 37%, very similar to the 42% prevalence observed in our sample of AIs living in South Dakota.

Second, our sample size was relatively small compared to many studies of HPV prevalence. Even so, this is the first study to examine HPV prevalence patterns in a high-risk AI population with a geographically localized White comparison group, and despite the small sample size, we were able to detect statistically significant differences between the groups, with important clinical and public health implications.

Third, we cannot disentangle the effects of urban residence from race. However, very few White women live on reservations, and only a negligible proportion of AI women were evaluated in the regionally-matched urban clinic, making the selection of samples evenly distributed between race and region virtually impossible.

Fourth, we did not use prior receipt of Gardasil vaccination as an exclusion criterion. Nevertheless, we are confident that our results remain unaffected by this limitation, since data on all White women and many AI women were collected before vaccine approval. Further, the AI women who were sampled in 2007-2008 were older than the target age range for HPV vaccination and had no insurance coverage from the Indian Health Service for this purpose. Even in the unlikely event that a few AI participants had prior vaccination, we would expect any resulting bias to be in the direction of less prevalent infection in this group.

Fifth, the absence of HPV vaccination among study participants prevented us from considering population differences in vaccine uptake; future research should examine this phenomenon more closely in minority communities.

Finally, we did not consider the additional prevention of HPV-31 provided by the Cervarix vaccine. Nevertheless, only four AI women, and no White women, were infected by HPV-31, so the inclusion of Cervarix in this analysis would have added confusion without altering our conclusions.

## Conclusions

This is the first study of HPV genotyping in a community-based sample of AI and White women. Our results suggest the existence of substantial differences in HPV prevalence and infection patterns among AI and White populations, highlighting the need for research on HPV in high-risk minority groups. If confirmed by future studies, our results might help to explain the documented cervical cancer disparities experienced by AI women. As it is, our findings underscore the need to evaluate vaccine efficacy in this population.

## List of Abbreviations

AI: American Indian; CI: confidence interval; EDTA: ethylenediaminetetraacetic acid; HPV: human papillomavirus; PCR: polymerase chain reaction.

## Competing interests

DSG reports that he is a member of the speakers' bureaus for Merck and GlaxoSmithKline, which pay him to give talks on HPV vaccination. None of the other authors have any commercial or other association that might pose a conflict of interest.

## Authors' contributions

DCSG participated in the design and coordination of the study, carried out the molecular genetic studies, collected patient samples, and assisted in drafting the manuscript. MCB participated in the design and coordination of the study and collected patient samples. CJM performed the statistical analyses and assisted in drafting the manuscript. DMM collected patient samples and participated in the data analysis. SCC assisted with interpretation. DSB participated in coordinating the study, supervised the overall conduct of the study, and assisted in drafting the manuscript. All authors read and approved the final manuscript.

## Pre-publication history

The pre-publication history for this paper can be accessed here:

http://www.biomedcentral.com/1471-2334/11/252/prepub

## References

[B1] Boyle P Global burden of cancer Lancet 1997 349 Suppl 2 SII23 6 9164444 10.1016/s0140-6736(97)90017-9

[B2] Band PR Gallagher RP Threlfall WJ Hislop TG Deschamps M Smith J Rate of death from cervical cancer among native Indian women in British Columbia CMAJ 1992 147 1802 1804 1458421 PMC1336656

[B3] Becker TM Wheeler CM Key CR Samet JM Cervical cancer incidence and mortality in New Mexico's Hispanics, American Indians, and non-Hispanic whites West J Med 1992 156 376 379 1574879 PMC1003275

[B4] Becker TM Wheeler CM McGough NS Jordan SW Dorin M Miller J Cervical papillomavirus infection and cervical dysplasia in Hispanic, Native American, and non-Hispanic white women in New Mexico Am J Public Health 1991 81 582 586 10.2105/AJPH.81.5.582 1849706 PMC1405076

[B5] Davidson M Schnitzer PG Bulkow LR Parkinson AJ Schloss ML Fitzgerald MA Knight JA Murphy CM Kiviat NB Toomey KE Reeves WC Schmid DS Stamm WE The prevalence of cervical infection with human papillomaviruses and cervical dysplasia in Alaska Native women J Infect Dis 1994 169 792 800 10.1093/infdis/169.4.792 8133094

[B6] Cobb N Paisano RE Cancer mortality among American Indians and Alaska Natives in the United States: regional differences in Indian health, 1989-1993 1997 Rockville, MD: Indian Health Service IHS publication no. 97-615-23

[B7] Frisch LL Allen GD Padonu G Dontje KJ Burhansstipanov L Social influences on Pap smear screening frequency Alaska Med 2000 42 41 45 47 10916857

[B8] Leman RF Espey D Cobb N Invasive cervical cancer among American Indian women in the Northern Plains, 1994-1998: incidence, mortality, and missed opportunities Public Health Rep 2005 120 283 287 16134569 10.1177/003335490512000311 PMC1497716

[B9] Espey DK Wu XC Swan J Annual report to the nation on the status of cancer, 1975-2004, featuring cancer in American Indians and Alaska Natives Cancer 2007 110 2119 2152 10.1002/cncr.23044 17939129

[B10] Risendal B Dezapien J Fowler B Papenfuss M Giuliano A Cancer prevention among urban southwestern American Indian women: comparison to selected Year 2000 national health objectives Ann Epidemiol 1999 9 383 390 10.1016/S1047-2797(99)00009-5 10475538

[B11] Risendal B DeZapien J Fowler B Papenfuss M Giuliano A Pap smear screening among urban Southwestern American Indian women Prev Med 1999 29 510 518 10.1006/pmed.1999.0565 10600432

[B12] Burhansstipanov L Dresser C Native American Monograph No.1: Documentation of the Cancer Research Needs of American Indians and Alaska Natives 1994 Washington, D.C.: National Institutes of Health

[B13] Burhansstipanov L Cancer among elder Native Americans. Continuing Education Module 1996 Denver, CO: Native Elder Health Care Resource Center

[B14] Thompson TG Grim CW Hartz GJ Smith PL Paisano EL Regional Differences in Indian Health, 2000-2001 2001 Indian Health Service

[B15] Horner MJ Ries LAG Krapcho M SEER Cancer Statistics Review, 1975-2006 2009 Bethesda, MD: National Cancer Institute

[B16] Bell MC Schmidt-Grimminger D Patrick S Ryschon T Linz L Chauhan SC There is a high prevalence of human papillomavirus infection in American Indian women of the Northern Plains Gynecol Oncol 2007 107 236 241 10.1016/j.ygyno.2007.06.007 17659767 PMC2396448

[B17] Datta SD Koutsky LA Ratelle S Human papillomavirus infection and cervical cytology in women screened for cervical cancer in the United States, 2003-2005 Ann Intern Med 2008 148 493 500 18378945 10.7326/0003-4819-148-7-200804010-00004

[B18] Dunne EF Unger ER Sternberg M Prevalence of HPV infection among females in the United States JAMA 2007 297 813 819 10.1001/jama.297.8.813 17327523

[B19] Hoskins WJ Perez CA Young RC Principles and Practice of Gynecologic Oncology 2000 3 Philadelphia: Lippincott Williams and Wilkins

[B20] South Dakota Tribal Government Relations Oglala Sioux Tribe 2004 http://www.state.sd.us/oia/oglala.asp Vol. 2009 Available at Accessed 14 August 2009

[B21] South Dakota Tribal Government Relations Rosebud Sioux Tribe 2004 http://www.state.sd.us/oia/rosebud.asp Vol. 2009 Available at Accessed 14 August 2009

[B22] Schwartz S The Arrogance of Ignorance: Hidden Away, Out of Sight and Out of Mind 2006 Brighton, Colorado: Native American Journalists Association

[B23] Ramirez R Rosebud Sioux Education Department Reduces Truancy Among Tribal Secondary Students Native American Rights Fund Legal Review 1999 24 http://www.narf.org/pubs/nlr/nlr24-2.htm Available at Accessed 22 February 2011

[B24] Sperling's Best Places Sioux Falls Metro Area, South Dakota 2009 http://www.bestplaces.net/Metro/Sioux_Falls-South_Dakota.aspx Available at Accessed 14 August 2009

[B25] Sioux Falls Municipal Government Sioux Falls: Population Profile 2009 Advameg, Inc http://www.city-data.com/us-cities/The-Midwest/Sioux-Falls-Population-Profile.html Available at Accessed 14 August 2009

[B26] Earth Day Network Urban Environment Report: Sioux Falls, South Dakota 2006 http://www.futuresiouxfalls.com/Competitive%20Assessment-FINAL.pdf Available at Accessed 4 November 2011

[B27] Markowitz LE Dunne EF Saraiya M Lawson HW Chesson H Unger ER Quadrivalent Human Papillomavirus Vaccine: Recommendations of the Advisory Committee on Immunization Practices (ACIP) MMWR Recomm Rep 2007 56 RR-2 1 24 17380109

[B28] Bosch FX Burchell AN Schiffman M Giuliano AR de Sanjose S Bruni L Tortolero-Luna G Kjaer SK Munoz N Epidemiology and natural history of human papillomavirus infections and type-specific implications in cervical neoplasia Vaccine 2008 26 Suppl 10 K1 16 18847553 10.1016/j.vaccine.2008.05.064

[B29] Khan MJ Castle PE Lorincz AT Wacholder S Sherman M Scott DR Rush BB Glass AG Schiffman M The elevated 10-year risk of cervical precancer and cancer in women with human papillomavirus (HPV) type 16 or 18 and the possible utility of type-specific HPV testing in clinical practice J Natl Cancer Inst 2005 97 1072 1079 10.1093/jnci/dji187 16030305

[B30] Jenkins D A review of cross-protection against oncogenic HPV by an HPV-16/18 AS04-adjuvanted cervical cancer vaccine: importance of virological and clinical endpoints and implications for mass vaccination in cervical cancer prevention Gynecol Oncol 2008 110 S18 25 10.1016/j.ygyno.2008.06.027 18653221

[B31] StataCorp Stata Statistical Software: Release 10 2007 College Sation, TX: StataCorp LP

[B32] Munoz N Bosch FX de Sanjose S Herrero R Castellsague X Shah KV Snijders PJ Meijer CJ International Agency for Research on Cancer Multicenter Cervical Cancer Study Group Epidemiologic classification of human papillomavirus types associated with cervical cancer N Engl J Med 2003 348 518 527 10.1056/NEJMoa021641 12571259

[B33] Bosch FX Manos MM Munoz N Sherman M Jansen AM Peto J Schiffman MH Moreno V Kurman R Shah KV Prevalence of human papillomavirus in cervical cancer: a worldwide perspective. International biological study on cervical cancer (IBSCC) Study Group J Natl Cancer Inst 1995 87 796 802 10.1093/jnci/87.11.796 7791229

[B34] Beller U Abu-Rustum NR Cervical cancers after human papillomavirus vaccination Obstet Gynecol 2009 113 550 552 19155953 10.1097/AOG.0b013e318191a54a

[B35] South Dakota Department of Health 2009 All Women Count! http://GetScreened.SD.gov/count Available at Accessed 14 August 2009

[B36] Benard V Lee NC Piper M Richardson L Race-specific results of Papanicolaou testing and the rate of cervical neoplasia in the National Breast and Cervical Cancer Early Detection Program, 1991-1998 (United States) Cancer Causes Control 2001 12 61 68 10.1023/A:1008959019019 11227926

